# Highly Graphitized Straw-Derived Carbon via Molten Salt Electrolysis for Potassium-Ion Batteries

**DOI:** 10.3390/ma18214877

**Published:** 2025-10-24

**Authors:** Yao Chang, Xinrui Wang, Yi Lu, Shijie Li, Zhenghao Pu, Wei-Li Song, Dongbai Sun

**Affiliations:** 1 Institute of Advanced Structure Technology, Beijing Institute of Technology, Beijing 100081, China; 3220232675@bit.edu.cn (Y.C.); weilis@bit.edu.cn (W.-L.S.); 2 State Key Laboratory of Advanced Metallurgy, University of Science and Technology Beijing, Beijing 100083, China; d202210671@xs.ustb.edu.cn (X.W.); d202310672@xs.ustb.edu.cn (Y.L.); sli@ustb.edu.cn (S.L.); 3 Department of Materials Science, Tohoku University, 6-6-02, Aramaki-Aza-Aoba, Aobo-ku, Sendai 980-8579, Japan; 4 Southern Marine Science and Engineering Guangdong Laboratory (Zhuhai), Zhuhai 519082, China

**Keywords:** molten salt electrolysis, biomass-derived graphitized carbon, potassium-ion batteries, energy storage

## Abstract

The conversion of straw biomass into highly graphitized carbon materials is achieved through an efficient molten salt electrolysis process at moderate temperatures (900–950 °C). Increasing the electrolysis temperature significantly enhances the degree of graphitization, structural ordering, and heteroatom removal efficiency, as evidenced by multiscale characterization and electrochemical simulations. The resulting graphitic material exhibits a highly ordered layered structure with improved crystallinity and a larger specific surface area. When used as a potassium-ion battery anode, this biomass-derived carbon delivers a reversible capacity of 232.9 mA·h·g^−1^ after 100 cycles and retains 230.8 mA·h·g^−1^ after 500 cycles, owing to its well-developed graphite framework, which accommodates volume changes and facilitates rapid ion diffusion. This study presents a sustainable and scalable strategy for transforming low-cost agricultural waste into high-performance energy storage materials and provides valuable insights into the electrochemical graphitization process.

## 1. Introduction

With the continuous advancement of global industrialization and the ongoing expansion of the human population, the demand for energy and materials has increased sharply. However, the reliance on traditional fossil fuels and non-renewable mineral resources not only exacerbates energy supply crises but also contributes to global challenges such as greenhouse gas accumulation, environmental pollution, and ecosystem degradation [1,2]. In this context, exploring sustainable, green, and low-carbon development pathways, along with the development of new high-performance materials and renewable energy technologies, has become a core priority in global scientific and industrial strategies.

As the only renewable carbon source, biomass resources are characterized by abundant reserves, wide distribution, carbon neutrality, and environmental friendliness. They are extensively applied in areas such as soil remediation, wastewater treatment, climate change mitigation, and clean energy production [3]. Among these, crop straw is one of the most abundant agricultural by-products, with an annual global output reaching billions of tons [4]. Nonetheless, most straw is still disposed of through open-field burning, landfilling, or low-value, extensive utilization. These practices result in significant waste of valuable carbon resources and produce substantial smoke and harmful gases during combustion, contributing to smog formation and posing serious threats to air quality and public health [5,6,7]. Therefore, achieving high-value, resource-efficient utilization of low-value, high-volume agricultural waste like straw is crucial for promoting sustainable agriculture, ecological protection, and the circular economy [8,9,10].

In this context, graphitization technology offers a promising solution. Graphite is a layered crystalline material composed of sp^2^-hybridized carbon atoms, which exhibits excellent electrical conductivity, thermal conductivity, chemical stability, and mechanical strength. It is widely used in applications such as lithium-ion battery anodes, supercapacitor electrodes, thermal management materials, and high-performance composites [11,12,13]. Conventional graphite is mainly sourced from natural graphite mining or produced from fossil-based precursors like petroleum coke and coal pitch via high-temperature heat treatment (typically above 2500 °C) [14,15,16]. These methods are limited by mineral resource constraints, high energy consumption, high costs, and significant carbon emissions, making them increasingly incompatible with green and low-carbon development principles. Recently, molten salt electrochemistry has emerged as a novel approach for efficient graphitization at relatively low temperatures. This method enables the structural ordering of carbon materials at temperatures as low as 800–900 °C. Tu et al. performed the graphitization of amorphous carbon in a CaCl_2_–LiCl melt at 700–850 °C, and the results indicated that higher electrolysis voltage, elevated temperature, and longer electrolysis time were more favorable for the transformation of the amorphous phase into the graphite phase [17]. Additionally, cathodic polarization facilitates the effective removal of heteroatoms, thereby enhancing the degree of graphitization and purity of the final product [18]. Studies by Jin et al. demonstrated that cathodic polarization in molten CaCl_2_ at 820 °C could transform carbon black into highly graphitized carbon. The polarization process plays a critical role in removing surface oxygen groups and promoting the long-range rearrangement of carbon atoms [19,20].

Motivated by recent reviews that highlight rapid progress and remaining challenges for high-performance K-ion anodes [21,22,23], we build upon previous feasibility studies by using renewable straw biomass and systematically treating electrolysis temperature as the primary tunable parameter to control structural ordering and heteroatom removal, while quantitatively linking these structural metrics to potassium-ion storage performance [2,3,4,5,6]. This study proposes a strategy to convert straw into highly graphitized, low-defect carbon materials via molten salt electrolysis. The process begins with pyrolysis of straw into amorphous carbon. Subsequently, in a typical electrochemical setup, the carbonized straw serves as the cathode, while a graphite rod functions as the anode. Molten CaCl_2_ was chosen as the electrolyte because it offers a moderate melting point (~772 °C), high ionic conductivity, and a wide electrochemical window that suppresses metal deposition while enabling rapid deoxygenation and ash removal via oxide ion (O^2−^) transport; electrolysis was then conducted under varied polarization conditions to systematically investigate the graphitization behavior. The products are comprehensively characterized using multiple analytical techniques. Based on the electrochemical parameters obtained, a multiscale modeling approach is developed to study the evolution of multi-physical fields at the electrode scale and their influence on the electrochemical graphitization kinetics. Finally, the synthesized graphitized material is evaluated as an anode for potassium-ion batteries, demonstrating stable cycling performance and significant reversible capacity. By coupling multiscale characterization with electrochemical simulations, we elucidate the structure–performance relationship for K^+^ storage and provide a tunable pathway to state-of-the-art potassium-ion anodes.

## 2. Experimental Section

### 2.1. Preparation of Template–Based Structural Carbon Precursors

The typical method for converting straw into carbon materials involves pyrolytic carbonization. In a representative procedure, 1.0 g of straw powder was carbonized in a tubular furnace at 700 °C under flowing Ar (99.9%) at 100 sccm for 2 h; the Ar purge was started 30 min before heating and maintained during heating and cool-down to ensure an oxygen-free environment. The heating rate was maintained at 5 °C·min^−1^ to produce a fluffy, straw-derived carbon intermediate. After carbonization, the resulting carbonized straw, containing various heteroatomic functional groups, was collected. Subsequently, 0.8 g of this carbonized straw was compressed into a pellet with a diameter of 1.5 cm under a pressure of 10 MPa. The pellet was then wrapped with a nickel mesh, and a molybdenum wire was wound around the assembly to serve as the current collector, forming the cathode for subsequent electrolysis.

### 2.2. Producing Carbon Nanomaterials Using Electrochemistry

Electrochemical graphitization of straw-derived carbon was systematically carried out in an alumina tubular reactor under a continuous high–purity argon atmosphere. Approximately 110 g of anhydrous calcium chloride was contained within an alumina crucible and initially dehydrated at 200 °C for 6 h to eliminate trace moisture. The temperature was subsequently raised to the target values of 900 or 950 °C under an inert environment. Constant–voltage electrolysis was implemented at 2.8 V (~300 mA, ~500 mA cm^−2^) for a duration of 8 h within a two-electrode system, utilizing a graphite rod as the anode and a compacted straw-derived carbon pellet as the cathode. This is because a shorter electrolysis time leads to incomplete graphitization, while a relatively longer electrolysis duration is beneficial for the transformation from amorphous carbon to ordered graphite. It should be noted that no catalytic additives were employed in these experiments. Under cathodic polarization, electrochemically driven removal of heteroatomic functional groups took place, thereby reducing structural disorder and promoting the reordering of carbon atoms into an extended graphitic framework. This process facilitated the development of a more crystalline carbon architecture. Following electrolysis, the solid products were retrieved from the solidified salt, subjected to washing with dilute hydrochloric acid to remove residual electrolytes, rinsed extensively with deionized water, and finally dried at 150 °C. The graphitized materials produced at 900 and 950 °C under identical electrical parameters were comparatively analyzed to assess the influence of temperature on the extent of graphitization and microstructural evolution.

### 2.3. Electrochemical Measurements

The working electrode was fabricated from the as-prepared carbon material, with potassium metal and a Whatman GF/D glass fiber (Whatman Ltd., Maidstone, UK) separator serving as the counter electrode and separator, respectively, to prevent internal short circuits. An electrolyte solution of 3.89 M potassium bis(fluorosulfuryl)imide (KFSI) in 1, 2–dimethoxyethane (DME) was employed. A homogeneous slurry was prepared by mixing the synthesized carbon nanomaterials, Super P carbon black, and sodium carboxymethyl cellulose (CMC) in an 8:1:1 mass ratio, followed by the addition of 0.8 mL N–methyl–2–pyrrolidone (NMP) and continuous stirring for 6 h. This slurry was then doctor–bladed onto a copper foil substrate and dried at 80 °C for 12 h. The dried electrode film was punched into 12 mm diameter disks, achieving an active material mass loading of approximately 1.5 mg cm^−2^. All cell assembly steps were carried out in an argon-filled glove box with oxygen and moisture levels maintained below 0.1 ppm. Electrochemical characterization was performed via cyclic voltammetry (CV) at a scan rate of 0.5 mV s^−1^ between 0.01 and 3.0 V (vs. K/K^+^) and galvanostatic charge/discharge (GCD) cycling within the same voltage window.

### 2.4. Characterization

The synthesized products were systematically characterized using a suite of analytical techniques. Crystal structure was determined by X-ray diffraction (XRD, Rigaku, Tokyo, Japan) using Cu Kα radiation. Raman spectroscopy was performed on a LabRAM HR Evolution system (HORIBA, Montpellier, France) with a 532 nm excitation laser. X-ray photoelectron spectroscopy (XPS) measurements were conducted at the 4B9A beamline of the Beijing Synchrotron Radiation Facility (Beijing, China) under ambient conditions to analyze the elemental composition and valence states. Morphological and microstructural observations were carried out using field emission scanning electron microscopy (FE–SEM, JEOL JSM–6701F, Tokyo, Japan) equipped with an energy-dispersive X-ray spectroscopy (EDS) detector (Thermo Fisher Scientific, Waltham, MA, USA), and transmission electron microscopy (TEM, JEOL JEM–2010, Tokyo, Japan). Specific surface area was determined from nitrogen adsorption data via the Braunauer–Emmett–Teller (BET) method, while pore size distribution was evaluated using the Barrett–Joyner–Halenda (BJH) model based on the adsorption branches of the isotherms. Nitrogen adsorption–desorption measurements were performed at 77.35 K using an Autosorb IQ volumetric adsorption analyzer (Quantachrome, Boynton Beach, FL, USA). XRD, XPS and other characterization tests were conducted twice to ensure the reliability of the data.

### 2.5. Computer Calculation

The Nernst–Planck equation in COMSOL Multiphysics 6.1 simulation is given as follows [24]:

(1) $$J_{i}=-D_{i}\nabla c_{i}-z_{i}u_{m,i}Fc_{i}\nabla \varphi_{i}$$

(2) $$\sum_{i}z_{i}c_{i}=0;$$

(3) $$u_{m,i}=\frac{D_{i}}{RT}$$

where *J_i_* (mol m^−2^ s^−1^) is the reaction flux of the ionic species, *D_i_* (m^2^ s^−1^) is the diffusion coefficient, *c_i_* (mol m^−3^) is the concentration of reactive ions, *z_i_* is the valence, *u_m,i_* (m^2^ V^−1^ s^−1^) is the mobility, *F* is the Faraday constant, and *φ_i_* (V) is the electrolyte potential.

The simulations were performed using a two-electrode setup, in which carbonized straw served as the cathode and a graphite rod was employed as the anode. The molten salt system was operated at temperatures of 1173.15 and 1223.15 K. The ion diffusion coefficient in the electrolyte was set to 2.55 × 10^−9^ cm^2^ s^−1^, with an initial concentration of 1.0 M. All parameters were based on experimental measurements. The boundary conditions were implemented and the system was numerically solved using COMSOL Multiphysics 6.1.

## 3. Results and Discussion

**Figure 1:**
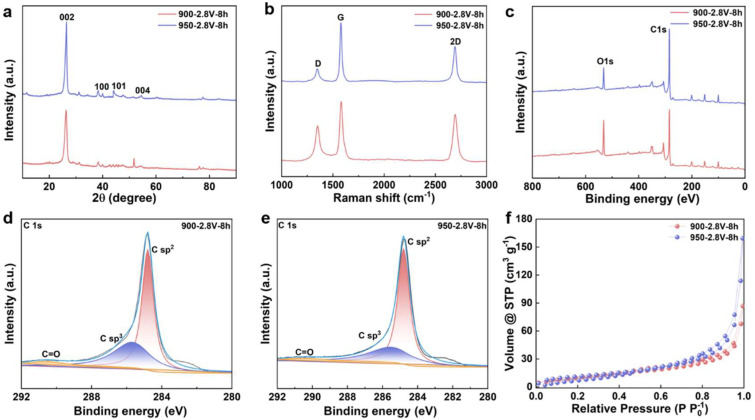
**Characterization of Straw-Derived Biochar Following Graphitization.** (**a**) XRD patterns; (**b**) Raman spectra; (**c**) full spectra of electrolysis products at 900 and 950 °C; (**d**) C 1s high-resolution XPS spectrum of electrolysis product at 900 °C; (**e**) C 1s high-resolution XPS spectrum of electrolysis product at 950 °C; (**f**) N_2_ adsorption–desorption isotherms of electrolysis products at 900 and 950 °C.

Figure S1 shows a schematic representation of biomass-to-graphite conversion via molten salt electrolysis at 900–950 °C, demonstrating the transformation of agricultural straw into layered graphitic materials through electrochemical carbonization and graphitization. The graphitization behavior of carbonized straw materials under different electrolysis temperatures was systematically characterized using XRD technology. Figure 1a displays the XRD patterns of the products obtained by electrolysis at 900 and 950 °C under a voltage of 2.8 V for 8 h. The results reveal that the products synthesized at both temperatures exhibit distinct diffraction peaks at 26.56°, 42.38°, 44.54° and 54.59°, which correspond to the (002), (100), (101) and (004) crystal planes of graphitic carbon (JCPDS No. 01–0646), respectively. It is particularly noteworthy that the intensity of the (002) diffraction peak shows a significant enhancement with increasing electrolysis temperature, indicating that elevated temperature effectively promotes the graphitization degree and improves the crystalline structure of the carbon materials.

To quantitatively investigate the influence of electrolysis temperature on the graphitization extent, the graphitization degree of the electrolytic products was accurately calculated based on the Mering–Marie equation and the Bragg equation [25,26,27]. As shown in Figure S2, under a constant cell voltage of 2.8 V, the graphitization degree of the products increased markedly from 37.44% at 900 °C to 45.33% at 950 °C. These calculation results further confirm that the rise in electrolysis temperature significantly facilitates the electrochemical transformation of amorphous carbon into highly crystalline graphitic carbon. The temperature elevation not only provides higher reaction activation energy but also promotes the rearrangement of carbon atoms and the formation of a graphite lattice, thereby enhancing the crystallinity and graphitization degree of the final product.

Raman spectroscopy was utilized to examine the graphitization behavior of carbon materials derived from straw. As illustrated in Figure 1b, the electrolyzed products displayed two characteristic peaks at approximately 1350 and 1580 cm^−1^, which correspond to the D band (associated with disordered carbon structures resulting from sp^3^-hybridized carbon) and the G band (attributed to the bond stretching vibration of sp^2^-hybridized carbon within the graphite lattice), respectively. At an electrolysis temperature of 900 °C, prolonging the electrolysis time led to a gradual decrease in the intensity of the D band, accompanied by a concurrent increase in the intensity of the G band. The emergence of a 2D band around 2700 cm^−1^ indicated the formation of a highly ordered graphitic structure in specific regions. When the temperature was elevated to 950 °C, the intensity of the D band became significantly lower than that of the G band, reflecting an enhancement in the structural ordering and degree of graphitization of the carbon material. The intensity ratio of the D to G bands (*I_D_/I_G_*) was employed to quantitatively assess the graphitization level; a lower value signifies improved crystallinity. As shown in Figure S3, when the electrolysis temperature increased from 900 to 950 °C, the *I_D_/I_G_* value narrowed from 0.627 to 0.279, clearly indicating the significant influence of elevated temperature on promoting the graphitization of carbon materials.

The XPS was utilized to investigate the electrochemical graphitization behavior of straw–derived carbon materials at varying electrolysis temperatures. As illustrated in Figure 1c, two distinct characteristic peaks were identified at binding energies of 284.8 eV and 532.4 eV, corresponding to the C1s and O1s electronic signals, respectively. Further analysis of the high–resolution C1s spectrum (Figure 1d,e) revealed that the electrolysis products could be deconvoluted into three sub–peaks: the peak at 284.8 eV was assigned to sp^2^–hybridized graphitic carbon, the peak at 285.5 eV was attributed to sp^3^–hybridized defective carbon, and the peak at 291.4 eV corresponded to C=O functional groups. As the electrolysis temperature increased, the intensity of the C=O-related characteristic peak gradually diminished, indicating the effective removal of oxygen–containing functional groups under cathodic polarization. Quantitative analysis of the oxygen content in the post–electrolysis materials demonstrated that the deoxygenation process plays a critical role in graphitization. After 8 h of electrolysis at 900 °C, the oxygen content of the product was 7.75 wt%, when the temperature was raised to 950 °C, the oxygen content significantly dropped to 3.25 wt%, confirming that elevated temperatures markedly enhance the degree of graphitization. These findings indicate that increasing the electrolysis temperature accelerates the deoxygenation process, thereby promoting the transformation of straw–derived carbon materials into highly graphitic structures.

To investigate the effect of reaction temperature on the specific surface area and pore structure of the products, N_2_ adsorption–desorption isotherms and corresponding pore size distributions were measured using a N_2_ physisorption analyzer. As shown in Figure 1f, two samples exhibit typical Type IV isotherms with distinct hysteresis loops in the relative pressure range of 0.4 < P/P_0_ < 1.0, indicating the presence of mesopores or macropores. With increasing temperature, the hysteresis loops of the other samples become less pronounced, reflecting variations in their pore structural characteristics. Pore size distributions were calculated using the Density Functional Theory (DFT) model, revealing that the pore sizes of three samples primarily fall within the 2–9 nm range (Figure S4a,b), further confirming the presence of significant mesoporous structures. The specific surface area (SSA) was determined via the Braunauer–Emmett–Teller (BET) method. The SSAs of two representative samples are 179 m^2^·g^−1^ and 148 m^2^·g^−1^, respectively. The change in SSA with rising electrolysis temperature can be attributed to enhanced rearrangement of carbon atoms into a more developed porous structure. The larger surface area provides additional adsorption sites for electrolyte ions, thereby improving the energy storage performance of the material.

**Figure 2:**
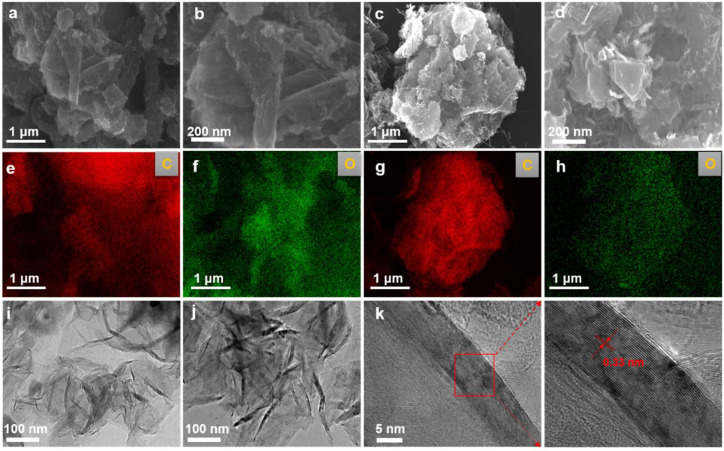
**Microstructural evolution of electrolytically graphitized samples.** (**a**,**b**) SEM micrographs depicting the morphological features of the sample synthesized at 900 °C and 2.8 V for 8 h; (**c**,**d**) corresponding SEM images of the product obtained under identical electrolysis conditions at an elevated temperature of 950 °C; (**e**,**f**) EDS elemental mappings of the sample electrolyzed at 900 °C; (**g**,**h**) EDS compositional profiles of the sample produced at 950 °C; (**i**) representative TEM micrograph of the sample prepared at 900 °C and 2.8 V for 8 h; (**j**) a TEM image illustrating the structural characteristics of the sample synthesized at 950 °C; (**k**) an HRTEM image revealing lattice fringes corresponding to graphitic ordering in the product formed at 950 °C and 2.8 V.

The morphological evolution of products obtained at different electrolysis temperatures was systematically characterized using FESEM and TEM. As shown in Figure 2a–d, at an electrolysis temperature of 900 °C, the product exhibited a bulk graphite morphology. When the temperature increased to 950 °C, the morphology gradually transformed into a more open, flake-like graphite structure. EDS mapping analysis of the samples (Figure 2e–h) indicated that they were primarily composed of carbon, with detectable traces of oxygen. The carbon originated from the graphite matrix itself, while the presence of oxygen suggests that some oxygen-containing functional groups remained after electrolysis. TEM analysis (Figure 2i,j) revealed that, with increasing temperature, the products evolved into thinner and more distinct graphitic nanoflakes. High-resolution TEM (HRTEM) of the sample obtained at 950 °C (Figure 2k) showed clear lattice fringes with an interlayer spacing of approximately 0.33 nm, corresponding to the (002) plane of graphite. These results are consistent with the standard graphite structure and indicate that the sample possesses high crystallinity.

**Figure 3:**
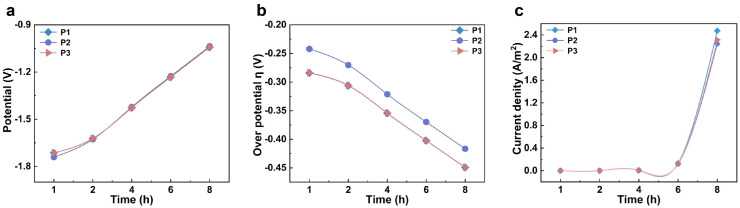
**The study of the cathodic deoxygenation process at the microscopic electrode scale.** (**a**) Time-dependent electrolyte potential profiles at three specific locations; (**b**) the temporal evolution of overpotential at three designated points; (**c**) chronological current density variations at three selected locations.

Using COMSOL finite element numerical simulation, this study investigated the influence of the relative positions of the anode and cathode on electrolyte potential distribution and electrochemical behavior. Based on the structural characteristics of carbonized products at different temperatures, a cathode surface model was constructed. Figure S5a,b present the simulation results of electrolyte potential on the cathode surface after applying a constant potential at 950 °C for 1 h and 8 h of electrolysis, respectively. The results show that, with increasing electrolysis time, the overall potential on the cathode surface shifts positively. During constant potential electrolysis, the voltage loss primarily compensates for cathodic polarization. Assuming stable system resistance, this results in a gradual positive shift in the cathode surface potential. To analyze the uniformity and evolution of the potential distribution, three characteristic points—P1, P2, and P3—were selected along the vertical axis of the cathode surface, corresponding to the top, middle, and bottom positions, respectively. As shown in Figure 3a, the electrolyte potential at each point increases with prolonged electrolysis time. Specifically, the potential at P1 changes from −1.714 V after 1 h to −1.043 V after 8 h; at P2 from −1.740 V to −1.037 V; and at P3 from −1.714 V to −1.042 V. These results indicate that the potential variation across different regions of the cathode surface remains relatively consistent throughout the electrolysis process, demonstrating good stability and uniformity in electrolyte potential distribution. The simulation outcomes align with experimental results, confirming that, under these temperature conditions, effective oxygen removal from carbonized straw is achievable, yielding a final product with uniform composition and a high degree of graphitization.

As shown in Figure S5c,d, the simulation results depict the distribution and evolution of the overpotential on the cathode surface. During the electrolysis process, the overpotential in the tip region of the electrode was significantly more negative than in other areas, indicating that the electrochemical deoxygenation reaction occurs preferentially at the tips. In the initial stage of electrolysis, the overpotential distribution across the entire cathode surface exhibited pronounced heterogeneity, which may be attributed to a relatively high concentration of initial defects within the carbon material. As electrolysis proceeded, the carbon framework underwent gradual rearrangement accompanied by continuous removal of heteroatoms. By 8 h of electrolysis, the overpotential distribution became more uniform, reflecting the evolution of the material structure and the adaptive optimization of electrochemical behavior. To further quantify the analysis, three characteristic points at the top, middle, and bottom of the cathode surface were selected (Figure 3b). At the start of electrolysis, the overpotentials at both the top and bottom points were approximately −0.284 V. Over time, these values shifted negatively, reaching about −0.449 V after 8 h. The variation trends at these two locations were consistent, with their curves almost overlapping, demonstrating good symmetry and synchrony. In contrast, the overpotential at the middle point remained more positive than at the two ends. However, as electrolysis continued, this difference gradually diminished, and eventually all three values converged. This indicates that the system progressively reached a uniform and stable electrolytic state, providing further evidence for the steady-state mechanism of structure–property co-evolution during the electrochemical deoxygenation process.

To further investigate the reaction mechanism and kinetic behavior during the electrochemical deoxygenation process, we simulated and analyzed the current density distribution on the cathode surface at 950 °C. Current density is a critical parameter that characterizes the rate and kinetics of electrochemical reactions. Figure S5e,f illustrate the current density distribution on the cathode surface after 1 h and 8 h of electrolysis, respectively. In the initial stage of electrolysis (1 h), the variation in current density was relatively small, indicating a low rate of heteroatom removal at this stage. This phenomenon is likely attributable to the high density of the cathode material, which was prepared through pressing. Figure 3c depicts the temporal variation in current density at three characteristic points on the cathode surface. During the first 4 h of electrolysis, the current densities at all three locations remained low with minimal variation. By 6 h, the current density began to increase significantly, reaching its maximum value at 8 h. This observation suggests that as electrolysis progresses, the consumption rate of the cathode reaction area gradually surpasses the decay rate of the current, resulting in an increase in current density. Furthermore, it implies that electrolysis at 950 °C effectively promotes the removal of heteroatoms and facilitates the formation of highly graphitized carbon materials.

**Figure 4:**
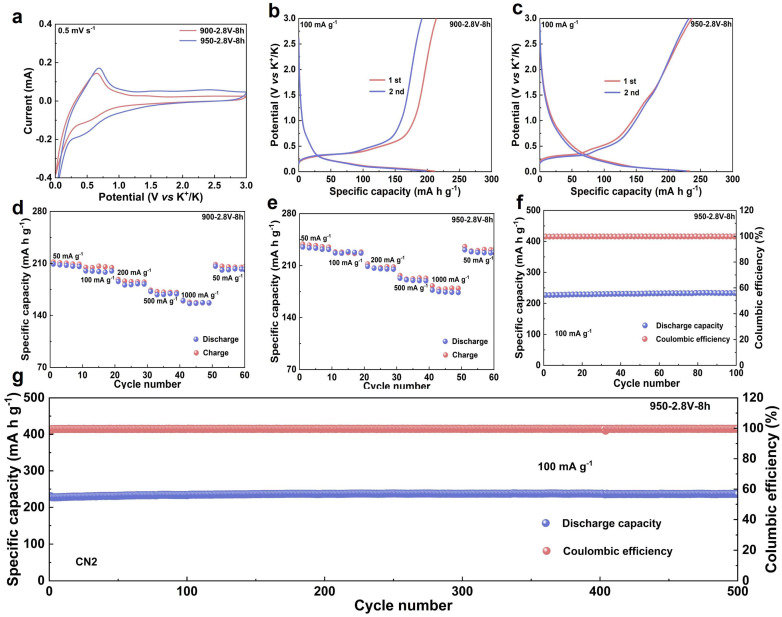
**Evaluation of the Electrochemical Performance of Materials.** (**a**) CV curves of the electrolytic product synthesized at 900/950 °C, measured at a scan rate of 0.1 mV s^−1^; (**b**) galvanostatic charge–discharge profiles of the 900 °C electrolytic product tested at a current density of 100 mA g^−1^; (**c**) charge–discharge voltage curves of the 950 °C product cycled at 100 mA g^−1^; (**d**) the rate capability of the sample fabricated at 900 °C under various current densities; (**e**) the rate performance of the 950 °C synthesized product evaluated at different current rates; (**f**) the cycling performance over 100 cycles at 100 mA g^−1^ for the product electrolyzed at 950 °C; (**g**) long-term cycling stability across 500 cycles at 100 mA g^−1^ for the material derived at 950 °C.

To evaluate the electrochemical performance of the synthesized materials, graphite obtained from heat treatment at 900 and 950 °C was systematically investigated as anodes for potassium-ion batteries (Table S2). Figure 4a presents the cyclic voltammetry (CV) curves of the two materials in half-cells within a voltage range of 0.01–3.0 V (vs. K^+^/K). A weak reduction peak observed around 0.5 V is associated with the decomposition of the electrolyte and the formation of a solid electrolyte interphase (SEI) on the electrode surface [28]. A sharp reduction peak near 0.06 V indicates the onset of potassium-ion intercalation into the graphite interlayers and the formation of potassium intercalation compounds. Correspondingly, a distinct oxidation peak appears around 0.63 V, which is attributed to the deintercalation of potassium ions from the graphite layers [29,30].

The energy storage performance was further assessed through galvanostatic charge–discharge (GCD) tests. At a current density of 100 mA·g^−1^, the first-cycle discharge specific capacities of the materials heat-treated at 900 and 950 °C were 211.4 and 233.2 mA·h·g^−1^, respectively (Figure 4b,c). The sample treated at 950 °C exhibited a higher reversible capacity compared to that treated at 900 °C, indicating that appropriately increasing the heat treatment temperature can enhance potassium storage performance. The rate capabilities of the two materials are presented in Figure 4d,e. At a current density of 50 mA·g^−1^, the sample treated at 950 °C delivered a discharge specific capacity of 234.9 mA·h·g^−1^. Even at a high current density of 1000 mA·g^−1^, it maintained a capacity of 174.5 mA·h·g^−1^, corresponding to a capacity retention of 74.29%. In contrast, the sample treated at 900 °C only delivered a discharge specific capacity of 209.1 mA·h·g^−1^ at 50 mA·g^−1^, suggesting a relatively limited potassium storage capability.

To further investigate the effect of a high degree of graphitization on the electrochemical stability of the electrodes, long-term cycling tests were conducted. The initial discharge specific capacity of the 900 °C sample was 203.3 mA·h·g^−1^, which declined slightly to 200.8 mA·h·g^−1^ after 100 cycles (Figures S6 and S7). Meanwhile, the 950 °C sample exhibited an initial discharge specific capacity of 226.8 mA·h·g^−1^, which increased slightly to 232.9 mA·h·g^−1^ after 100 cycles (Figure 4f), demonstrating excellent cycling stability. Moreover, after 500 cycles at a current density of 100 mA·g^−1^, this material still retained a reversible capacity of 230.8 mA·h·g^−1^ (Figure 4g), further confirming its remarkable long–term cycling performance. The superior electrochemical performance can be attributed to the high degree of graphitization; the highly ordered carbon layered structure not only provides effective buffer space to accommodate volume variation during potassium-ion insertion/extraction but also facilitates potassium-ion diffusion kinetics, significantly enhancing both potassium storage capacity and structural stability.

## 4. Conclusions

This study demonstrates the successful conversion of straw-derived carbon into highly graphitized materials through molten salt electrolysis at moderate temperatures. Increasing the electrolysis temperature from 900 to 950 °C significantly enhances the degree of graphitization, structural ordering, and deoxygenation efficiency. Multiscale characterization and simulation results reveal improved potential uniformity and reaction kinetics at elevated temperatures, which facilitate carbon atom rearrangement and the effective removal of heteroatoms. When utilized as an anode for potassium-ion batteries, the resulting graphitic material exhibits exceptional electrochemical performance, including a high reversible capacity of 232.9 mA·h·g^−1^ after 100 cycles and remarkable long-term stability, with 230.8 mA·h·g^−1^ retained after 500 cycles. These superior properties are attributed to the highly ordered graphitic structure, which accommodates volume variation during potassium-ion intercalation and deintercalation, thereby facilitating ion diffusion. This work provides a sustainable and scalable approach for valorizing biomass waste into high-performance energy storage materials, while also enhancing the understanding of the electrochemical graphitization mechanism and promoting the application of sustainable carbon materials in advanced batteries. Future work will employ operando XRD/Raman and controlled annealing studies to elucidate the order of porosity evolution and quantify its impact on rate capability and long-term stability.

## Data Availability

The original contributions presented in this study are included in the article/Supplementary Material. Further inquiries can be directed to the corresponding authors.

## Supplementary Materials

The following supporting information can be downloaded at https://www.mdpi.com/article/10.3390/ma18214877/s1: Figure S1: Highly graphitized straw-derived carbon prepared by molten salt electrolysis for potassium-ion batteries; Figure S2: The degree of graphitization of the product obtained at temperatures of 900 and 950 °C; Figure S3: The I_D_/I_G_ value of the product obtained at temperatures of 900 and 950 °C; Figure S4: Pore size distribution profiles of the electrolysis products obtained at 900 and 950 °C; Figure S5: Study of the cathodic deoxygenation process at the microscopic electrode scale. (a) Electrolyte potential distribution on the cathode surface after 1 h of electrolysis at 950 °C; (b) Electrolyte potential evolution on the cathode surface following 8 h of electrolysis; (c) Overpotential variation across the cathode surface after 1 h of electrolysis at 950 °C; (d) Overpotential distribution on the cathode surface subsequent to 8 h of electrolysis; (e) Current density mapping on the cathode surface after 1 h of electrolysis at 950 °C; (f) Current density distribution on the cathode surface after 8 h of electrolysis; Figure S6: Cycling performance over 100 cycles at 100 mA g^−1^ for the product electrolyzed at 900 °C; Figure S7: EIS data before cycling at different temperatures and after 10 cycles. (a) 900 °C; (b) 950 °C; Table S1: The establishment of COMSOL simulation parameters; Table S2: Comparative Overview of Carbon Anodes in Lithium/Sodium/Potassium Ion Batteries.
