# Supplementary material for: Highly Graphitized Straw-Derived Carbon via Molten Salt Electrolysis for Potassium-Ion Batteries
Source: Materials (Basel). 2025 Oct 24;18(21):4877. doi: 10.3390/ma18214877 (PMC12608522; doi:10.3390/ma18214877)
Supplement: Supplementary file 1 [file materials-18-04877-s001.zip › materials-3896925-supplementary.pdf]

Highly Graphitized Straw-Derived Carbon via Molten Salt Electrolysis for Potassium-Ion Batteries

Yao Chang [1], Xinrui Wang [2], Yi Lu [2], Shijie Li [2], Zhenghao Pu [3,*], Wei-Li Song [1] and Dongbai Sun [4,*]

[1] Institute of Advanced Structure Technology, Beijing Institute of Technology, Beijing 100081, China
[2] State Key Laboratory of Advanced Metallurgy, University of Science and Technology Beijing, Beijing 100083, China
[3] Department of Materials Science, Tohoku University, 6-6-02, Aramaki-Aza-Aoba, Aobo-ku, Sendai 980-8579, Japan
[4] Southern Marine Science and Engineering Guangdong Laboratory (Zhuhai), Zhuhai 519082, China
* Correspondence: pu.zhenghao.e8@tohoku.ac.jp (Z.P.); sundongbai@mail.sysu.edu.cn (D.S.)

Keywords: Molten salt electrolysis; Biomass-derived graphitized carbon; Potassium-ion batteries; Energy storage

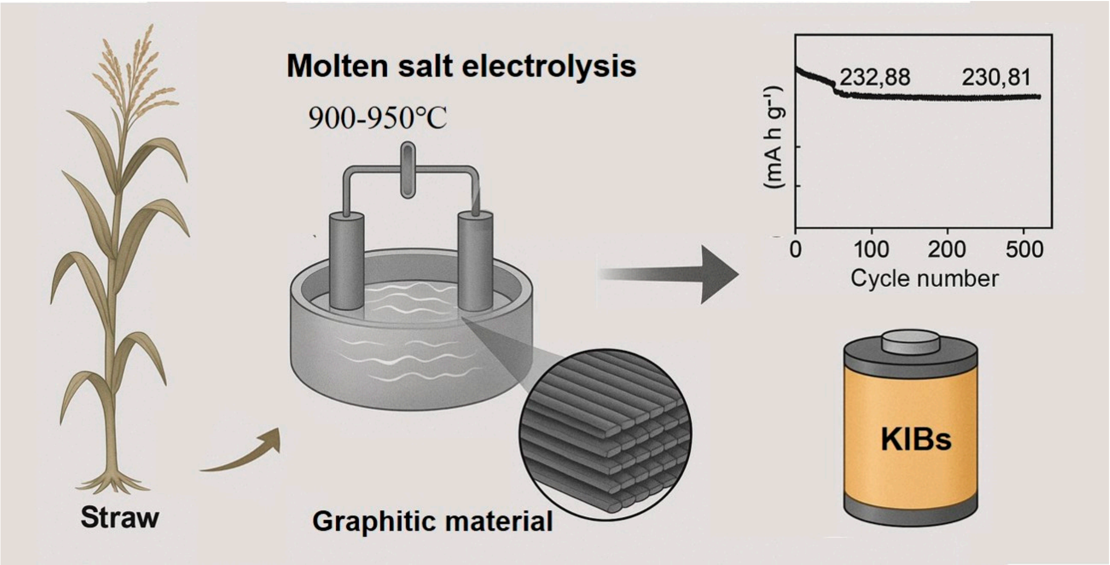

Figure S1. Highly graphitized straw-derived carbon prepared by molten salt electrolysis for potassium-ion batteries.

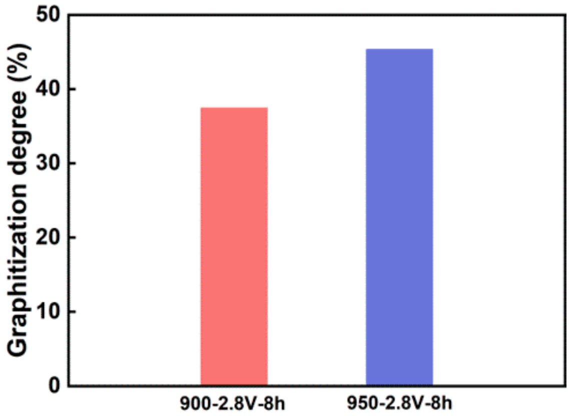

Figure S2. The degree of graphitization of the product obtained at temperatures of 900 and 950 °C.

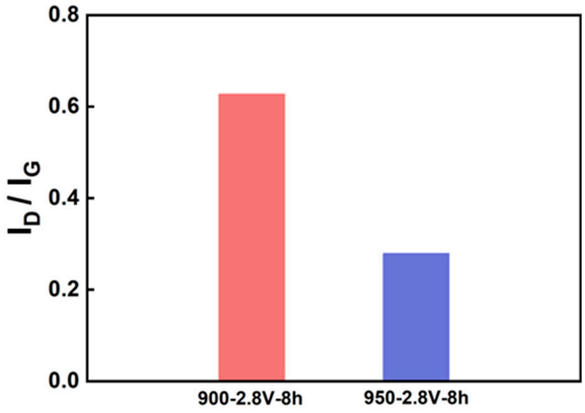

Figure S3. The I_D/I_G value of the product obtained at temperatures of 900 and 950°C.

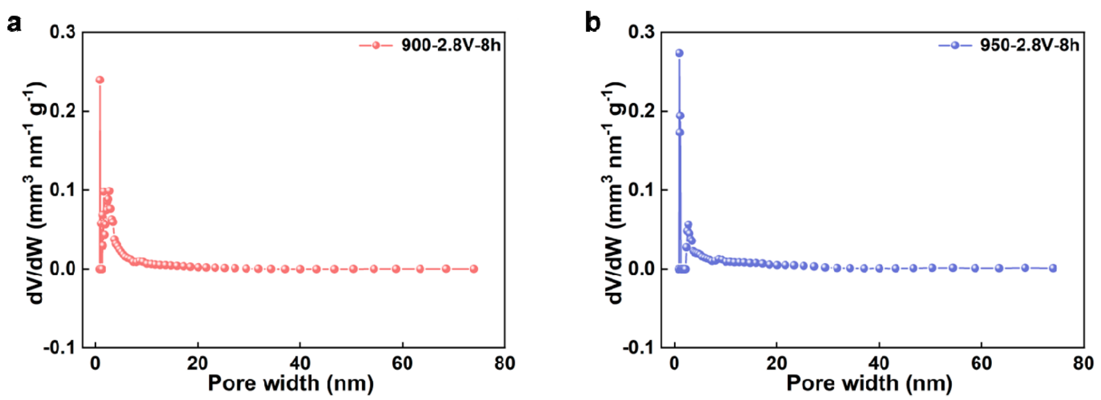

Figure S4. Pore size distribution profiles of the electrolysis products obtained at 900 and 950 °C.

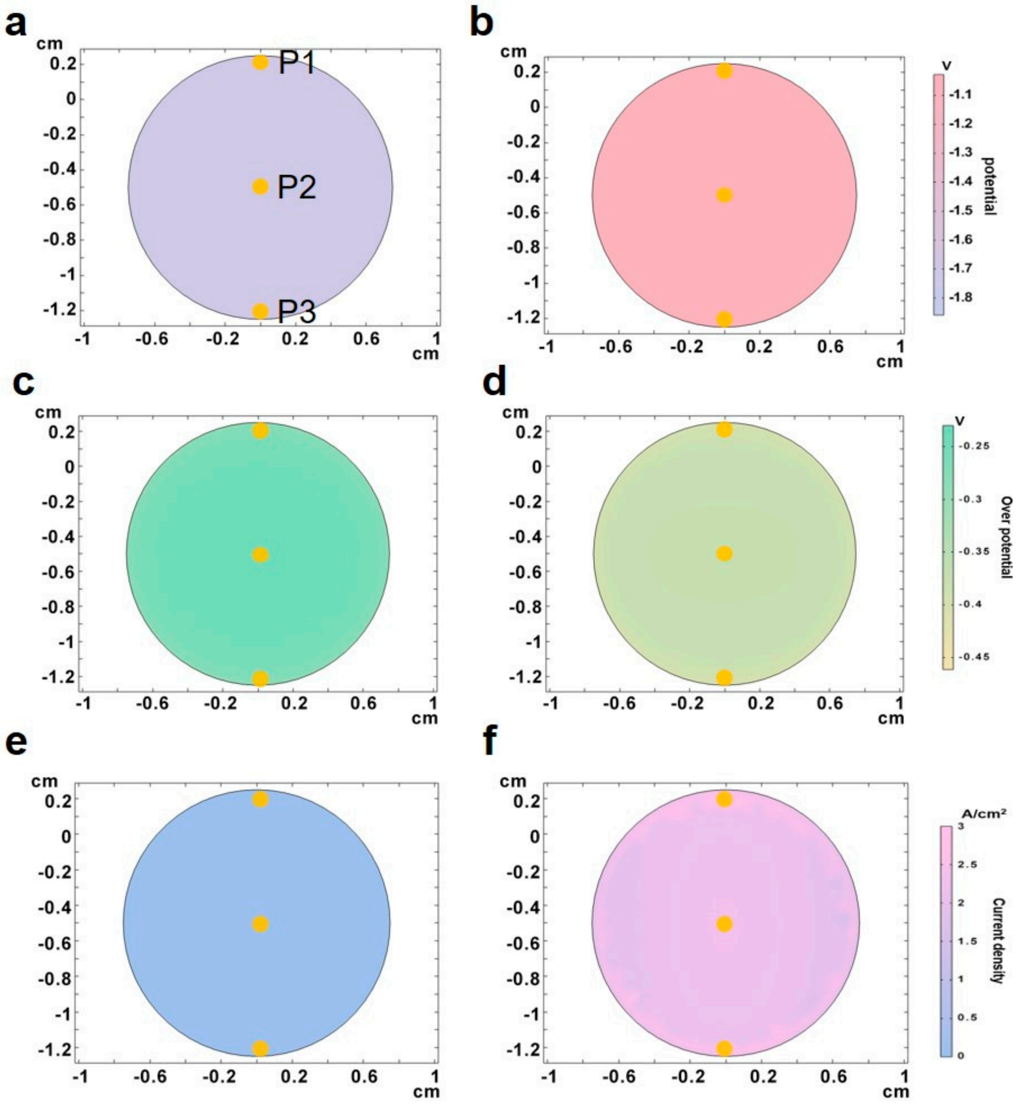

Figure S5. Study of the cathodic deoxygenation process at the microscopic electrode scale. (a) Electrolyte potential distribution on the cathode surface after 1 h of electrolysis at 950 ℃; (b) Electrolyte potential evolution on the cathode surface following 8 h of electrolysis; (c) Overpotential variation across the cathode surface after 1 h of electrolysis at 950 ℃; (d) Overpotential distribution on the cathode surface subsequent to 8 h of electrolysis; (e) Current density mapping on the cathode surface after 1 h of electrolysis at 950 ℃; (f) Current density distribution on the cathode surface after 8 h of electrolysis.

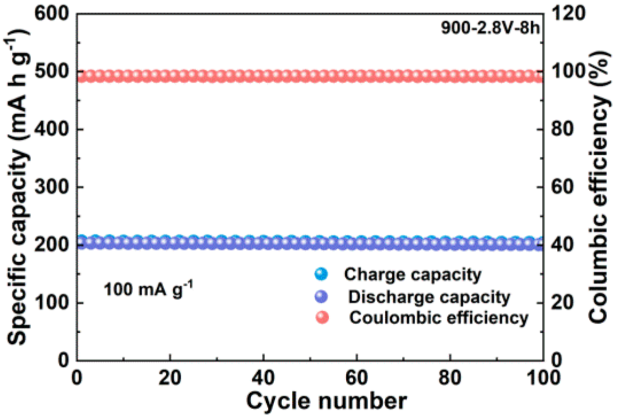

Figure S6. Cycling performance over 100 cycles at 100 mA g^{-1} for the product electrolyzed at 900 °C.

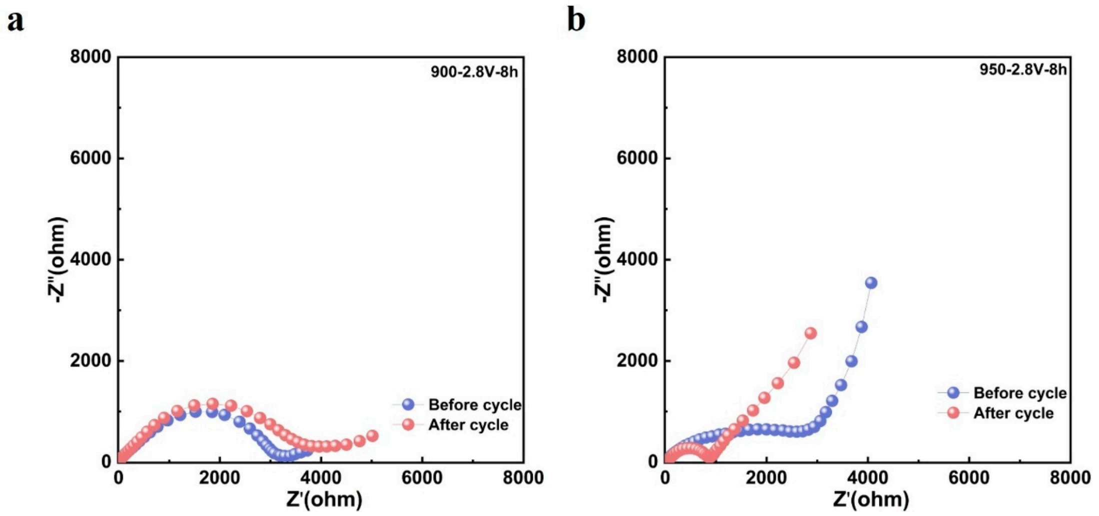

Figure S7. EIS data before cycling at different temperatures and after 10 cycles. (a) 900 ℃；(b) 950 ℃.

Table S1. The establishment of comsol simulation parameters.

**Designation**	**Numerical value**	**Unit**	**Data source**
Electrode voltage	2.8	V	Experimental parameters
Cathode standard equilibrium potential	2.876	V	Calculation
Anode standard equilibrium potential	0	V	Model setting
Electrolyte conductivity	2.25	S/m	Experimental parameters
Temperature	1223	K	Experimental parameters
Reference molar concentration	1	mol/L	nernst equation
Oxygen ion concentration	0.01572	mol/cm^3	Calculation
Diffusion coefficient of electrolyte ions	2.55×10^{-9}	$cm^2\ s^{-1}$	Experimental parameters

Table S2. Comparative Overview of Carbon Anodes in Lithium/Sodium/Potassium Ion Batteries

**Parameter**	**Li-ion (graphite)**	**Na-ion (hard carbon)**	**K-ion (graphitized carbon; this work context)**
Working potential *vs* M^+/M	~0.1 V (*vs* Li^+/Li)	~0.0 – 0.15 V (*vs* Na^+/Na)	~0.2 – 0.3 V (*vs* K^+/K)
Theoretical/practical capacity	372 mAh g^{-1} (LiC_6)	260 – 350 mAh g^{-1} (hard carbon, typical)	279 mAh g^{-1} (KC_8), practical depends on order/porosity
Dominant storage mechanism	Intercalation in graphite galleries (staging to LiC_6)	Pore filling + surface/defect adsorption; limited intercalation in graphite	Intercalation in graphite (KC_x staging) + minor surface/defect contribution
Ionic radius (Shannon, VI)	$Li^+ \approx 0.76$ Å	$Na^+ \approx 1.02$ Å	$K^+ \approx 1.38$ Å
Graphite compatibility	Fully compatible	Not compatible in carbonate electrolytes; needs hard carbon or ether co-intercalation	Compatible (forms KC_8)
Volume change (full insertion)	~10% (LiC_6)	typically <10 – 15% (hard carbon)	~60 – 70% (KC_8)
SEI behavior (carbonate)	Relatively stable with additives (e.g., FEC)	More fragile/soluble; additive-dependent	Fragile; improved with KFSI/KTFSI salts and tailored additives
Electrolyte notes	$LiPF_6$ in EC/EMC/DEC common	$NaPF_6/NaClO_4$ in EC/PC + additives	KFSI/KTFSI in EC/EMC/DEC; ethers also explored
Rate capability (typical)	High	Moderate (depends on pore structure)	Moderate; limited by expansion, improved by micro/meso-porosity and
